# Pathways to plant domestication in Southeast Anatolia based on new data from aceramic Neolithic Gusir Höyük

**DOI:** 10.1038/s41598-021-81757-9

**Published:** 2021-01-22

**Authors:** Ceren Kabukcu, Eleni Asouti, Nadja Pöllath, Joris Peters, Necmi Karul

**Affiliations:** 1 Department of Archaeology, Classics and Egyptology, University of Liverpool, Liverpool, L69 7WZ UK; 2 Staatliche Naturwissenschaftliche Sammlungen Bayerns, Staatssammlung für Anthropologie und Paläoanatomie München, Karolinenplatz 2a, 80333 Munich, Germany; 3 ArchaeoBioCenter, Institute of Palaeoanatomy, Domestication Research and the History of Veterinary Medicine, Ludwig-Maximilians-Universität München, 80539 Munich, Germany; 4 Department of Prehistory, Istanbul University, Istanbul, 34134 Turkey

**Keywords:** Plant domestication, Archaeology

## Abstract

Southeast Anatolia is home to some of the earliest and most spectacular Neolithic sites associated with the beginning of cultivation and herding in the Old World. In this article we present new archaeobotanical and zooarchaeological data from Gusir Höyük, an aceramic Neolithic habitation dating to the 12th-late 11th millennia cal BP. Our results show selective use of legume crop progenitors and nuts during the earlier part of this period, followed by the management of cereal and legume crop progenitors from the mid-11th millennium cal BP. This contrasts with data available from other Anatolian habitations indicating broad spectrum plant use with low crop progenitor inputs. Early aceramic Neolithic Anatolian plant and animal exploitation strategies were site-specific, reflecting distinctive identities and culinary choices rather than environmental constraints. A multivariate evaluation of wheat grain metrics alongside botanical and radiometric data indicate that early wheat domestication in southeast Anatolia occurred at a faster pace than predicted by current hypotheses for a protracted transition to farming in Southwest Asia. We argue that this phenomenon is best explained as a corollary of the increasing importance of cereals in feasting at southeast Anatolian sites characterised by increasing architectural complexity and elaboration during the 11th millennium cal BP.

## Introduction

**Figure 1:**
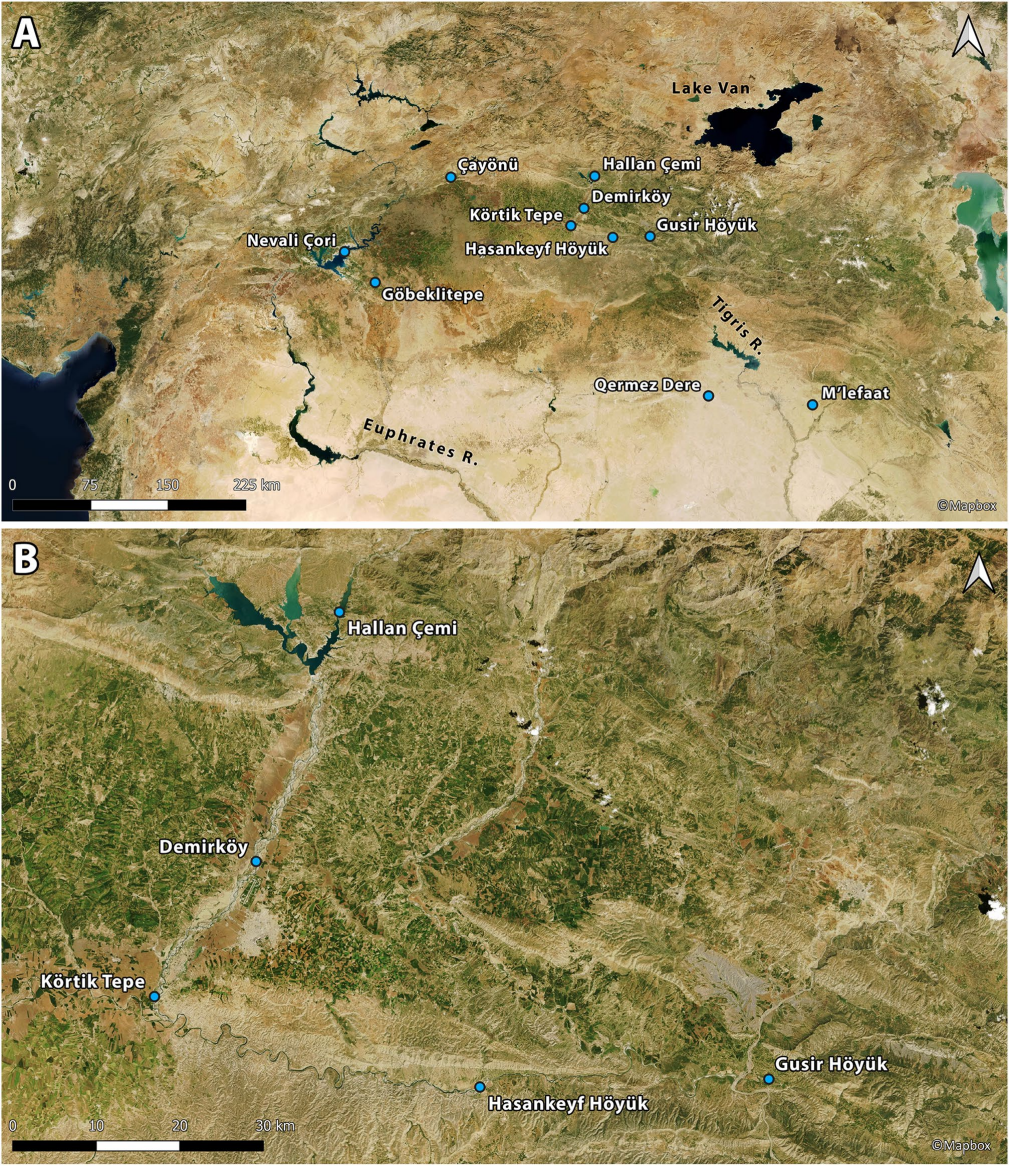
Satellite maps showing location of the archaeological sites mentioned in the text. **(A)** PPN sites in southeast Anatolia and northwestern Iraq; **(B)** PPN sites in the vicinity of Gusir Höyük. Maps created using QGIS 3.10 (free and open software) with tilesets available from Mapbox (CC BY).

Southeast Anatolia is home to some of the earliest Neolithic sites associated with the transition from foraging to farming in the Old World. Since the first excavations at Çayönü Tepesi in 1964 by the Joint Istanbul-Chicago Prehistoric Project, led by Halet Çambel and Robert Braidwood, archaeological fieldwork has revealed an impressive range of aceramic Neolithic sites spanning ~ 1500 years, from the mid 12th to the 10th millennia cal BP^1^ (Fig. 1). However, following nearly six decades of intensive fieldwork and spectacular archaeological discoveries, little is still known about the nature and context of the regional aceramic Neolithic plant management practices and the process of early crop domestication. To the east, in the Tigris basin, sites dated to the early aceramic/Pre-Pottery Neolithic A (PPNA) horizon (12th-early 11th millennia cal BP) sampled for archaeobotanical remains (Hallan Çemi, Körtik Tepe, Demirköy, Hasankeyf Höyük) have revealed no evidence for the intensive exploitation of crop progenitor species^2–4^. To the west, in the Euphrates basin, the only site from which some archaeobotanical materials have been published to date is Göbeklitepe which has also produced low densities of crop progenitor charred macro-remains but more abundant microbotanical (phytolith) remains^5,6^. Cereals and especially legumes have been found in much higher densities at sites with phases dated to the Early PPNB (EPPNB) (mid-late 11th millennium cal BP) such as Çayönü and Nevali Çori. Despite this abundance, indicators of phenotypic domestication at these sites remain scarce and ambiguous^7–10^.

The absence of evidence for the intensive exploitation of crop progenitor species in southeast Anatolia during the PPNA and its late onset in the EPPNB pose several challenges for our understanding of the evolution of plant cultivation and domestication in this region and, by implication, across the Fertile Crescent of Southwest Asia. The first two millennia of the Holocene (~ 11,700–10,500 cal BP) witnessed abrupt climatic improvement that caused the rapid expansion of grasslands across Southwest Asia^11,12^. That southeast Anatolia occupied a pivotal position in the primary zone of the distribution of crop progenitors is supported by multiple lines of evidence including genetic data, historical and modern vegetation surveys, and ethnobotanical studies^10,11,13,14^. However, unlike the situation observed in northern Syria and the Levant^15^ this landscape abundance is not reflected in the southeast Anatolian PPNA archaeobotanical assemblages^10,16^. Although the lack of large-scale sampling with the use of machine-assisted water flotation might explain, at least in part, the limited archaeobotanical recovery at some sites (e.g., Göbeklitepe), cereals are also rare at other sites (e.g., Hallan Çemi, Körtik Tepe) where comprehensive sampling has taken place. At the same time, cereal and legume crop progenitors are far more abundant at EPPNB sites characterised by architectural complexity and symbolic/ritual elaboration (e.g., Çayönü, Nevali Çori). Interestingly, these sites also contain some of the earliest evidence in Southwest Asia for intensified caprine and cattle management during this period^17^. This evidence brings to the fore two key questions: Could the low proportions of crop progenitors found at southeast Anatolian PPNA sites be explained as the result of incomplete sampling and/or archaeobotanically under-explored variation in the regional vegetation ecologies? Was the early use of cereal and legume crop progenitors linked to wider socio-cultural shifts manifested in the emergence of large and architecturally more complex sites straddling the PPNA-EPPNB horizon? In turn, these questions have wider significance, extending beyond the interpretation of the southeast Anatolian record, for understanding the historical process, context and proximate causes of the development of the earliest agricultural economies in Southwest Asia during the early Holocene^11^.

Gusir Höyük is one of only three excavated aceramic Neolithic sites in southeast Anatolia that preserve archaeological deposits dated from the PPNA through to the EPPNB horizons, the other two being Göbeklitepe and Çayönü. To date, it is also the only site of the three from which archaeobotanical remains have been retrieved from radiometrically dated archaeological contexts with the use of machine-assisted water flotation. Excavations conducted between 2009 and 2014 by an Istanbul University team under the direction of Necmi Karul, have unearthed a permanent habitation locale characterised by architectural elaboration and distinctive material culture assemblages that present affinities, as well as important differences, with materials excavated at other early PPN sites in the Anatolian Euphrates and Tigris basins^18^. Gusir Höyük thus provides a unique opportunity to explore these questions on the cultural and ecological context of crop progenitor use in early PPN southeast Anatolia through the analysis of primary archaeobotanical data obtained from adequately sampled archaeological contexts.

## Site context

**Figure 2:**
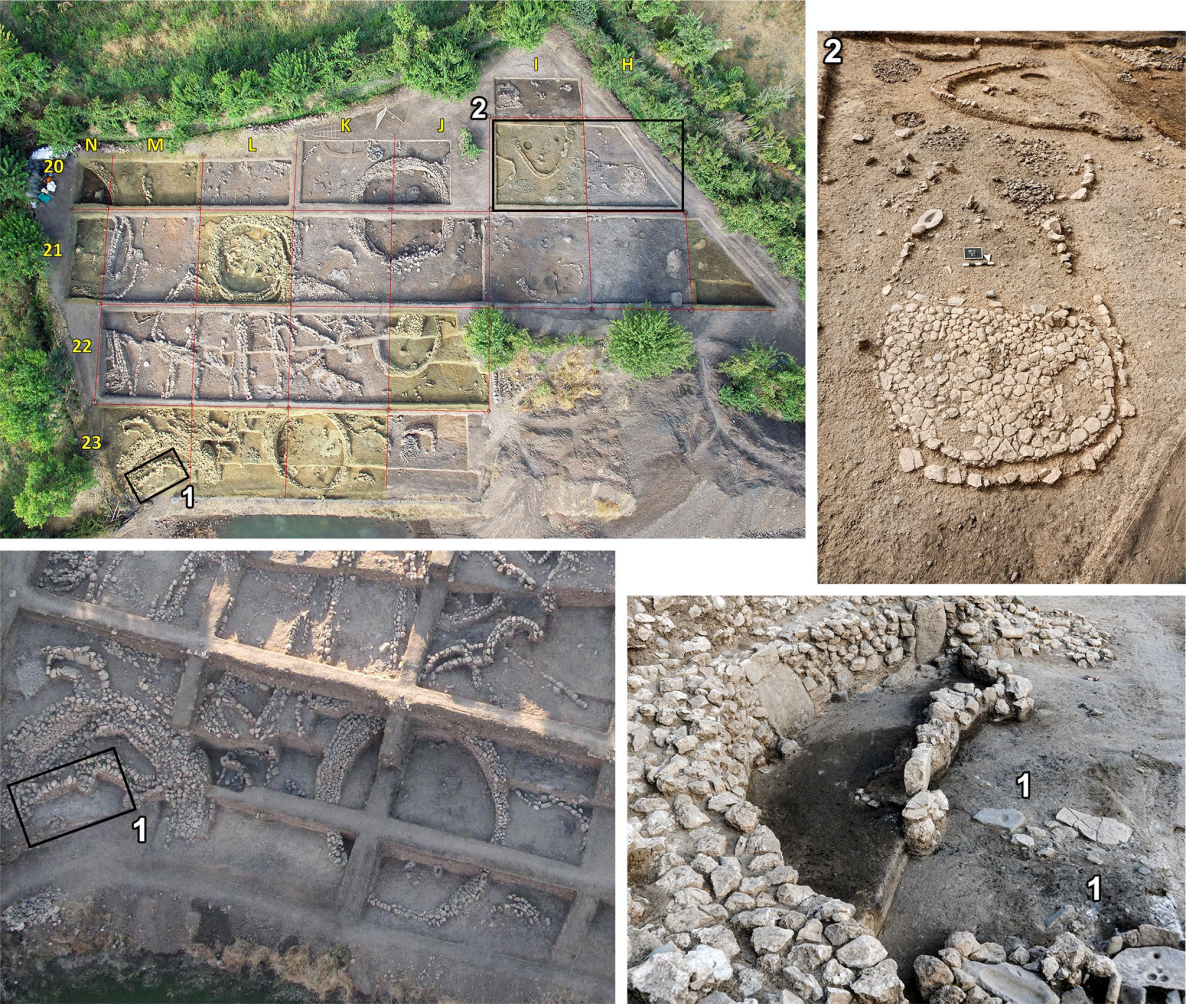
Top left: aerial view of the Gusir Höyük excavation grid (grid squares sampled for archaeobotanical remains highlighted in yellow). (1) Close-up views of square 23-M showing the location of the sampled mid-11th millennium cal BP contexts that yielded charred *Triticum* macro-remains; (2) close-up view of square 20-H with stone-paved oval structure (open at one end) and adjacent square 20-I with the late 11th millennium cal BP fire pits (photos from the Gusir Höyük project archive).

**Figure 3:**
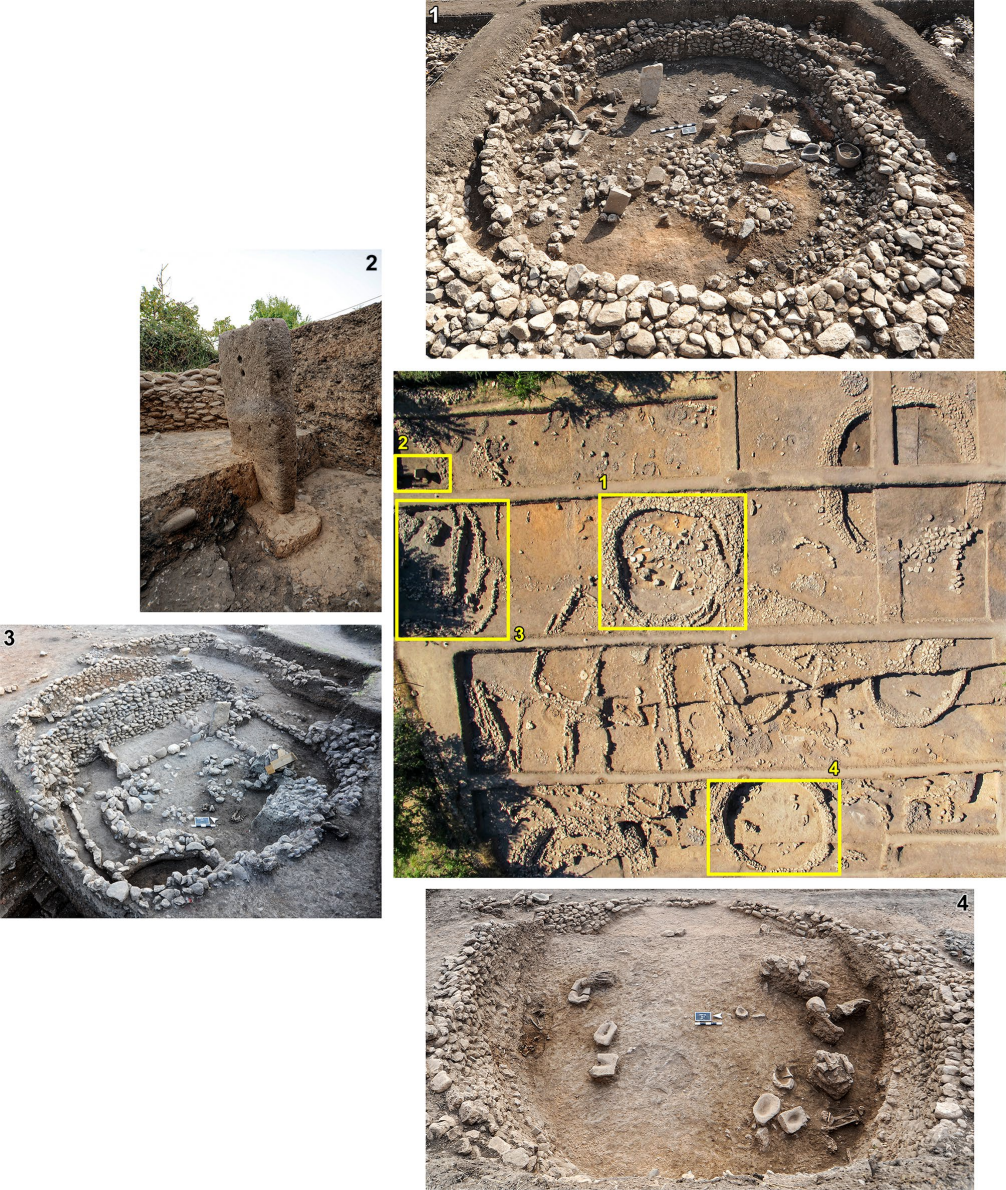
Characteristic examples of Gusir Höyük architecture (photos from the Gusir Höyük project archive).

Gusir Höyük (37°43′37.90" N, 41°49′16.25" E; ~ 535 m a.s.l.) is located in the Siirt province of southeast Turkey near the intersection of the Tigris River with its tributary Botan, on the western shore of Gusir lake a sinkhole fed by the Kavaközü stream and groundwater (Fig. 1 and Supplementary Fig. S1)^18,19^. Excavations have revealed an area of ~ 0.20 ha containing 7–8 m of PPN deposits rich in chipped stone and ground stone and the remains of stone-built structures (Figs. 2 and 3). The earliest excavated phases comprise semi-subterranean (~ 2 m deep) circular buildings with internally plastered walls, stone basins and centrally located monolithic pillars (1–1.5 m in height) sometimes associated with wild sheep and goat horncores. Their floors were renewed at least twice, with human burials placed beneath each floor layer. Stratigraphically later phases comprise rectangular buildings with rounded corners and sunken floors (as deep as 1 m below ground level) which cut into earlier circular structures. Some of these buildings were subdivided into additional rectangular units. Stone pillars were set at the centre of buildings (sometimes in pairs) or were irregularly placed at their corners, inside walls or in front of the walls. The stratigraphically latest excavated phases include less substantial oval-shaped buildings with one end open to provide access. These buildings had flat stone floors and were separated by large open spaces occasionally containing fire pits^18,19^.

This paper presents the results of the analysis of 36 archaeobotanical flotation samples from Gusir Höyük (averaging ~ 40–50 L of sediment per sampled context) derived from 10 excavation grid squares (Fig. 2). Most samples (n = 27) derived from building fill deposits radiocarbon dated to ~ 11,400–10,900 cal BP. The first results of zooarchaeological analysis from the same PPNA contexts are also presented. 9 samples were collected from a closely controlled set of deposits excavated in grid squares 23-M (ashy spreads from floors and between wall fills) and 20-I (fire pit fills) radiocarbon dated to ~ 10,500–10,300 cal BP (Fig. 2, Supplementary Tables S1-S2, Supplementary Data Files S1–S2). Excavations at Gusir Höyük did not reach virgin soil and the analyses of the site stratigraphy, chronology, architecture and other categories of archaeological finds are ongoing^19^. It follows that the radiometric dates reported in this paper reflect the chronology of the sampled deposits and should not be taken as representative of the site chronology as a whole.

## Results

### Archaeobotanical and zooarchaeological assemblage composition

**Figure 4:**
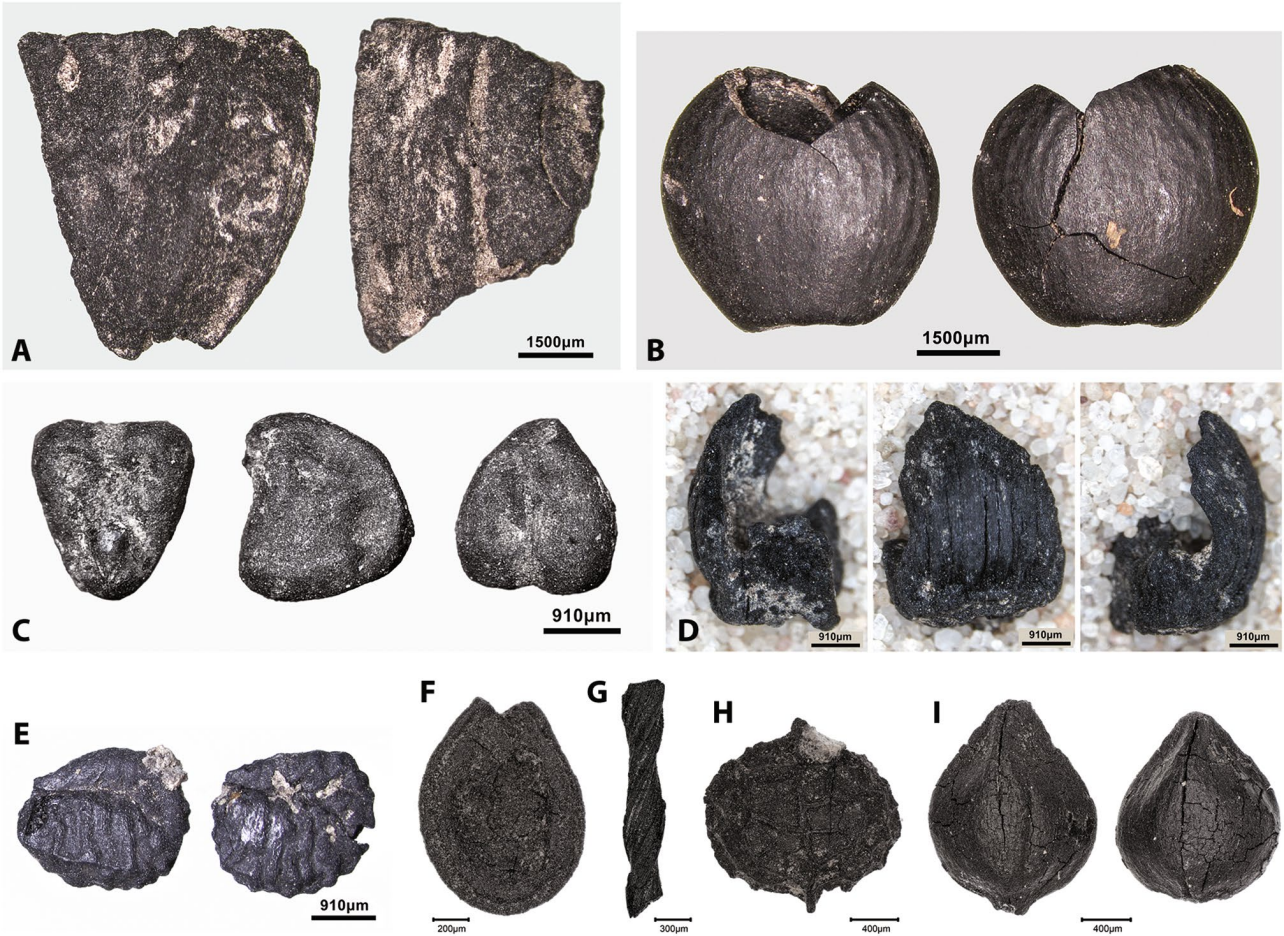
Charred plant macroremains from Gusir Höyük. **(A)** *Prunus* subg. *Amygdalus* nutshell; **(B)** *Pistacia* nutshell; **(C)** *Vicia ervilia* seed; **(D)** *Aegilops* chaff; **(E)** *Medicago radiata*; **(F)** *Solanum* sp.; **(G)** *Stipa* awn; **(H)** *Neslia* sp.; **(I)** Cyperaceae (photos by Ceren Kabukcu).

**Figure 5:**
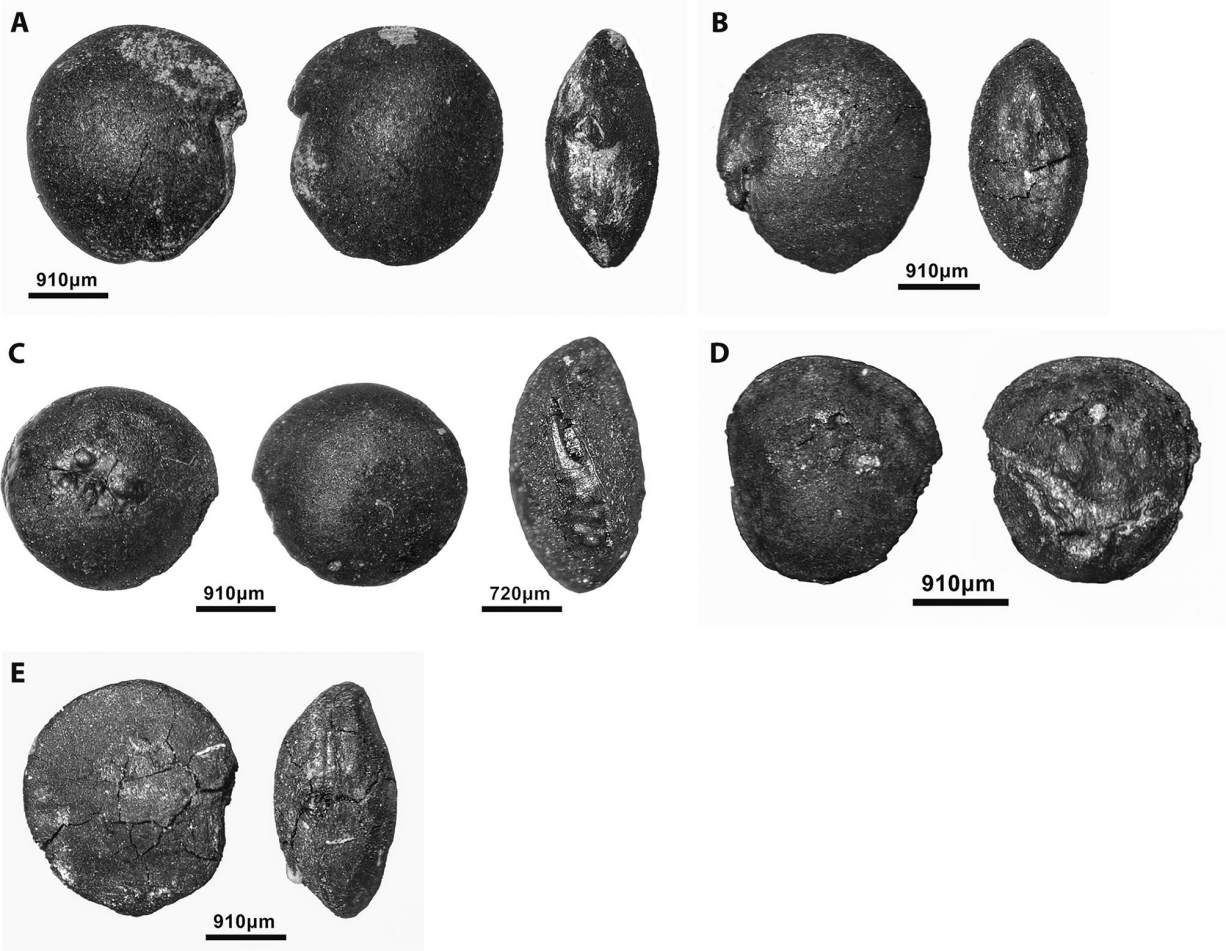
Gusir Höyük lentil specimens. **(A,B)** Well-preserved *Lens* specimens from PPNA contexts; **(C–E)** seeds showing soaking/cooking-related distortions of their cotyledons and testa (photos by Ceren Kabukcu).

The results of the analysis of the flotation samples grouped by chronological phase show clear diachronic shifts in the range and proportions of the charred plant taxa found in the archaeobotanical assemblage (Supplementary Table S1, Supplementary Data File S2). The PPNA samples (radiocarbon dated to ~ 11,400–10,900 cal BP) are dominated by *Prunus* subg. *Amydgalus* and *Pistacia* nutshell and legumes (*Lens*, *Vicia ervilia*, *Vicia/Lathyrus*, Vicieae and *Onobrychis*) (Figs. 4A–C and 5). Hackberry (*Celtis*) stones are very sporadically present. Grasses comprise small-seeded Poaceae, morphologically indistinguishable to genus or species level. Other wild taxa are dominated by Brassicaceae and Caryophyllaceae which occur in low frequencies. The very low representation of Cyperaceae indicates limited use of wetland plants during this period (Fig. 4I).

**Figure 6:**
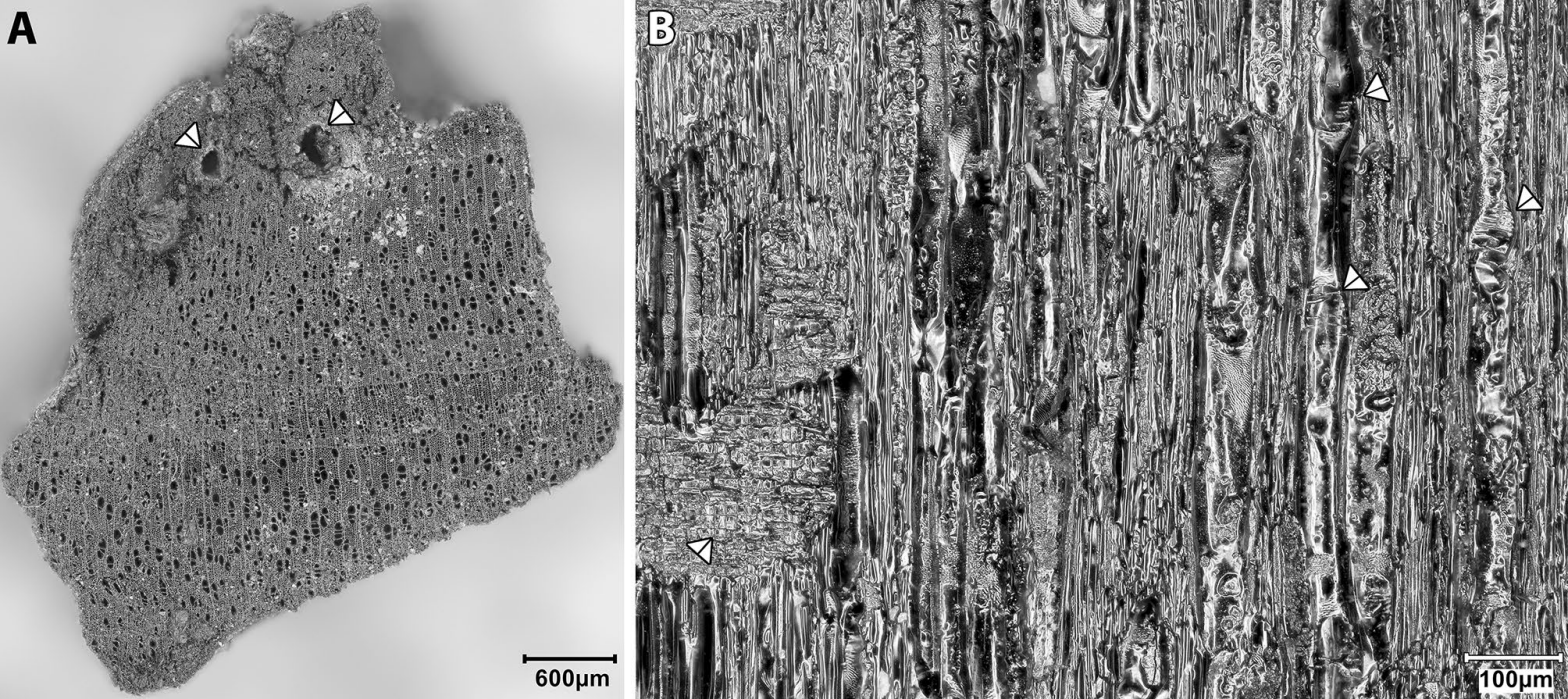
*Betula* (birch) charred wood from Gusir Höyük. **(A)** TS plane showing annual growth increments with boreholes; **(B)** RLS plane showing characteristic scalariform perforation plates (arrows to the right) and ray cell walls affected by fungal degradation (arrow to the left) (photos by Eleni Asouti).

Limited exploitation of riparian/wetland habitats is also indicated by the anthracological remains retrieved from the PPNA flotation samples (Supplementary Table S1, Supplementary Data File S2) that are overwhelmingly dominated by *Prunus* subg. *Amygdalus* and *Pistacia* wood charcoals. Other taxa (*Acer, Prunus, Rhamnus,* deciduous *Quercus*, *Celtis*) register very modest counts (< 10 fragments each) and may represent minor components of the local woodland vegetation. Riparian trees and shrubs are absent except for the very sporadic presence of *Fraxinus*. The sole non-local tree species is *Betula* (Fig. 6). *B. pendula* (syn. *B. verrucosa*) is rare in eastern Anatolia, found in subalpine vegetation on Mt. Ararat and Bingöl Dağ at elevations > 2500–2700 m a. s. l.^20,21^. All *Betula* charcoals found at Gusir Höyük preserve signs of wood degradation prior to burning, including fungal hyphae and/or boreholes. This suggests that at least some of the birch wood was collected from the banks of the nearby stream as dry driftwood, although *Betula* would have been more common in the landscape at the start of the Holocene^21,22^.

Further support for the preferential exploitation of dryland vegetation habitats is provided by the first results of zooarchaeological analysis of PPNA contexts, which show a distinctive record particularly for the avifauna (Supplementary Table S2). 99.1% of the bird bone belongs to partridges (*Alectoris chukar*, *Perdix perdix*). This is in stark contrast to other contemporary avifaunal assemblages in southeast Anatolia which are extremely diverse in terms of the species and habitats exploited, as exemplified by the Hallan Çemi bird bone assemblage^23^. Both partridge species prefer open and dry grassland habitats in rocky hills. Aquatic birds, which are dominant at all other PPNA sites with access to wetlands, are virtually absent from Gusir Höyük with the exception of 2 bones of a crane (*Grus grus*) and a white-tailed eagle (*Haliaaetus albicilla*). It appears therefore that fowling focused on habitats away from permanent watercourses. This is largely corroborated by the mammalian record, which is dominated by wild caprines^24^. That the inhabitants of Gusir Höyük did not completely ignore the potential of wetland and riparian habitats is evidenced by the finds of wild boar, fish and beaver bones as well as freshwater mussel shells in low frequencies.

**Figure 7:**
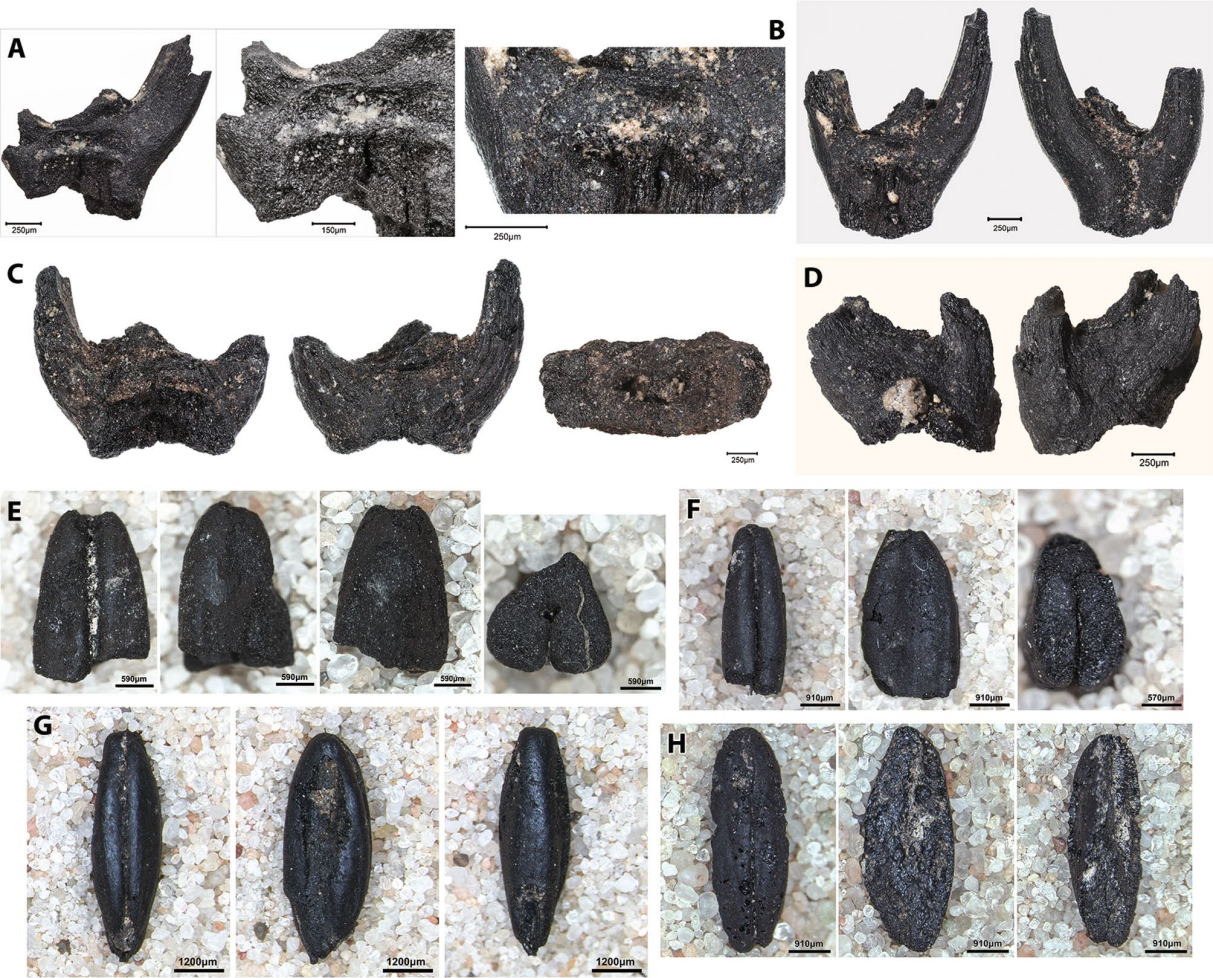
Charred *Triticum* spp. grain and chaff remains from square 23-M at Gusir Höyük. **(A)** *T. boeoticum* wild-type spikelet fork; **(B)** *T. boeoticum* wild-type spikelet fork (front & back view and close-up of front view showing partially preserved dehiscent scar; **(C)** *T. turgidum dicoccoides* spikelet fork (front, back and top views); **(D)** tetraploid wheat terminal spikelet; **(E)** partial grain of *T. turgidum dicoccoides* (apical end—left to right: ventral, lateral, dorsal & cross-section views); **(F)** partial grain of *T. boeoticum* (apical end—left to right: ventral, lateral & cross-section views); **(G)** *T. boeoticum* complete grain (left to right: ventral, lateral, dorsal views); **(H)** *T. boeoticum* complete grain (left to right: ventral, lateral & dorsal views) displaying testa lifting/splitting on the lateral and dorsal views, likely due to pounding damage (photos by Ceren Kabukcu).

The 4 flotation samples analysed from 23-M came from ashy spreads on plaster floors associated with architectural remains radiocarbon dated to ~ 10,500–10,300 cal BP, which cut into an earlier PPNA round structure (Fig. 2). This distinctive assemblage is dominated by a mixture of crop progenitor taxa including *Lens*, *Vicia* (*V. ervilia* and *Vicia/Lathyrus* type) and 1- and 2-grained einkorn (*Triticum boeoticum*) and emmer (*T. turgidum* ssp. *dicoccoides*) grain and chaff found alongside *Aegilops* grain and chaff, and small-seeded Poaceae (Figs. 4C,D, 5, 7). The presence of small-seeded legumes (*Medicago radiata*, *Astragalus/Trigonella* and Fabaceae indet.) and other taxa associated with ruderal habitats is also notable (Fig. 4E–H). Compared to the PPNA charred plant assemblage, these samples contained lower concentrations of *Prunus* subg. *Amygdalus* and *Pistacia* nutshell. An AMS radiocarbon determination on a single einkorn seed produced an estimated range of 10,500–10,308 cal BP (1σ) and 10,558–10,282 cal BP (2σ) with a median age of 10,422 cal BP (Supplementary Data File S1). Pending further analysis, this suggests that the deposits containing charred crop progenitor remains may date from as early as ~ 10,500 cal BP.

5 flotation samples were available for analysis from fire pit contexts excavated in 20-I radiocarbon dated to ~ 10,300 cal BP (Fig. 2, Supplementary Data Files S1-S2). Unlike the 23-M samples they do not contain cereals, which likely reflects the impact of context-related variation on taxon representation. *Lens*, *Vicia ervilia*, *Vicia/Lathyrus* and small-seeded Fabaceae are well-represented in these samples alongside *Prunus* subg. *Amygdalus* and *Pistacia* nutshell. The wetland signal is stronger in the 23-M and 20-I samples, which contain *Bolboschoenus/Carex* seeds and a single seed of wild vine (*Vitis sylvestris*) a species associated with riparian habitats. 23-M wood charcoals are dominated by *Fraxinus* (180 fragments) (Supplementary Table S1, Supplementary Data File S2). *Betula* driftwood is co-dominant (84 fragments) while *Quercus* and *Pistacia* are also present. By contrast, *Prunus* subg. *Amygdalus* is absent from 23-M and registers a modest count (15 fragments) in 20-I which is dominated by *Pistacia* charcoal (190 fragments). Together these data suggest that by the EPPNB (mid-late 11th millennium cal BP) fuel wood collection at Gusir Höyük targeted both dryland and riparian habitats. The exploitation of riparian habitats is also indicated by the exceptional presence of *Alnus* wood charcoal in 20-I. No archaeofaunal remains from EPPNB contexts have been analysed yet.

## Crop progenitor use and the status of crop relatives

The *Lens* spp. seeds retrieved from all sampled contexts at Gusir Höyük appear to be, at least morphometrically, of the wild type measuring on average 2.37 mm (with a range of 2.79–1.7 mm). This range agrees well with the measurements previously reported from Çayönü^7^ (Fig. 5). A significant proportion of complete seeds and cotyledon fragments displayed curving of the cotyledon and/or bulging under the testa (Fig. 5C–E) which suggest high moisture content and/or high temperature charring possibly in relation to cooking practices such as soaking^25^.

Determining the status of charred *Triticum* spp. as wild or domesticated has been the object of long-standing debate in the literature^26^. The most widely deployed indicator is the morphology of the attachment scar on the spikelet forks, denoting shattering habit. Observing this feature in charred archaeological specimens is often problematic due to the impact of crop processing practices. Grain dehusking by pounding the cereal ears often results in a ‘ripped’ section, removing most or all of the attachment scar, which inhibits identifying if the spikelets belong to dehiscent or indehiscent ears^27^. Most of the spikelet forks found at Gusir Höyük display similar dehusking damage (Fig. 7A). Only a few specimens preserve a small portion of the lower attachment scar, displaying the smooth break associated with wild-type dehiscent ears (Fig. 7B). Other potential domestication indicators include changes in seed morphology and size. Recent work on modern *Triticum* accessions suggests that changes in grain size involve multiple parameters that may vary between different species. For example, larger seed size in einkorn is more closely associated with a shortening of the grain length and a general widening of its breadth and height, alongside a reduction in the overall variability of grain measurements (i.e., a trend towards more standardized length, width and breadth dimensions)^28^. In addition, recent research on the morphometrics of modern charred and uncharred wheat grain has indicated that multivariate assessments of grain dimensions and morphology may also enable species- and/or local landrace-level identifications^29,30^.

**Figure 8:**
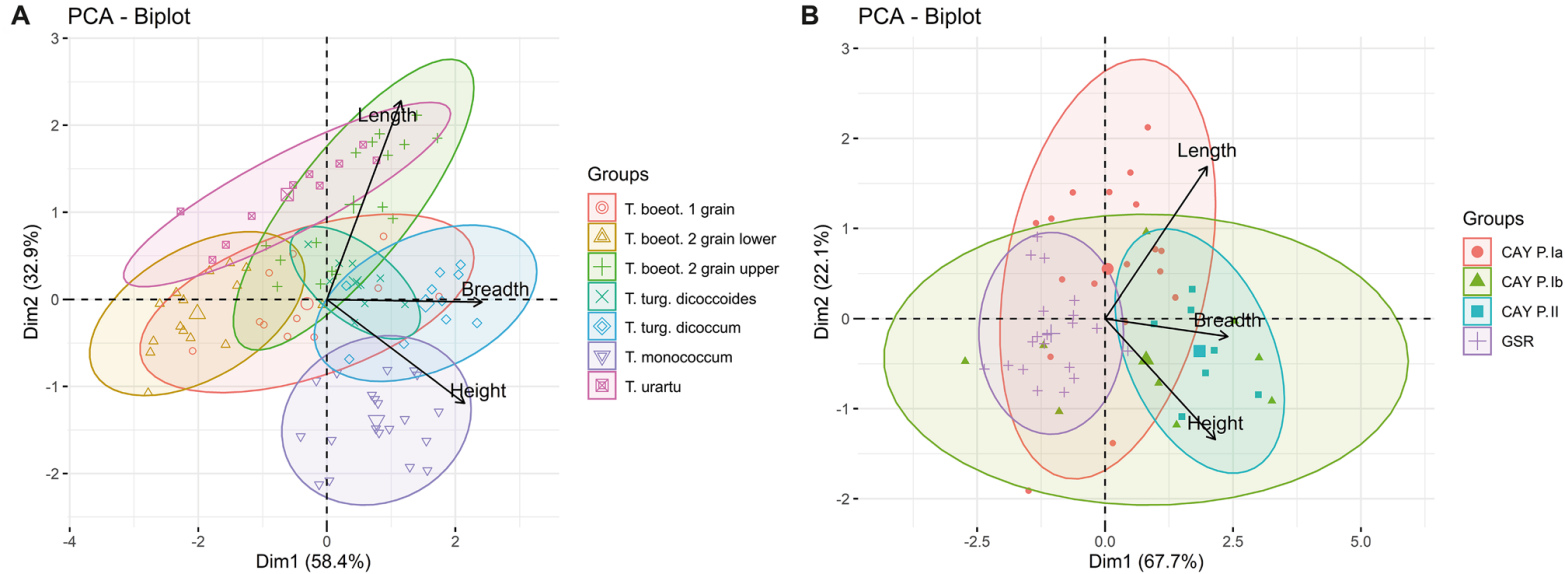
Principal component analysis of measurements on modern *Triticum* spp. accessions and archaeological charred grain. **(A)** PCA biplot of modern accession measurements; **(B)** PCA biplot of archaeological *Triticum* charred grain measurements (CAY = Çayönü; GSR = Gusir).

In order to evaluate *Triticum* grain size at Gusir Höyük we obtained measurements (length, breadth, height) from individual charred grain specimens and modern accession grains, which were analysed with Principal Component Analysis (PCA) (Fig. 8). The number of complete charred *Triticum* grains found at Gusir was low (7 in total). For this reason, we also obtained breadth and height measurements from incomplete specimens preserving either the apical or embryo end of the grain, which were used to impute the missing length measurements (a full description of the procedure is included in the Methods section below). We also included in this evaluation the metrics previously published from Çayönü, the sole other southeast Anatolian site from which individual charred wheat grain measurements are available covering both its aceramic and Pottery Neolithic (PN) phases^7^. To facilitate the detection of diachronic trends in the archaeological *Triticum* measurements dataset we assembled the published grain measurement data in three groups: ‘Phase Ia’ (Round, Grill and Channel Building sub-phases; > 8630–8245 cal BC), ‘Phase Ib’ (Cobble Paved and Cell Building sub-phases; ~ 8250–7350 cal BC) and ‘Phase II’ (PN; 7140–6820 cal BC)^31,32^. Measured modern wheat accessions include *T. boeoticum*, *T. urartu*, *T. turgidum* ssp. *dicoccoides*, *T. monococcum* and *T. turgidum* ssp. *dicoccum* sourced from the John Innes Centre Germplasm Resource Unit (GRU). The full set of measurements used in PCA is included in Supplementary Data Files S3-S5.

The PCA of the modern accession measurements provided a reference summary of the morphometric observations commonly reported in standard botanical descriptions of *Triticum* grain^33^. The PCA biplot (Fig. 8A) shows that breadth and height account for a greater proportion (58%) of the variation captured along Dimension 1 separating crop species (right-hand side of the plot) from the wild-type accessions (left-hand side). Both einkorn and emmer crop seeds are wider compared to the wild-type accessions. Length accounts for a lower proportion (32.9%) of the variation captured along Dimension 2 separating the longer wild-type accessions (upper portion of the plot) from the generally shorter crop species (mid and lower portions). PCA also enabled the visualization of finer qualitative distinctions reported in the literature^33^ by re-affirming that in 2-grained einkorn the grains from the lower portion of the floret are distinctly narrower than those from the upper portion of the floret (Fig. 8A).

When compared to the PCA biplot of the modern *Triticum* accessions, the Gusir/Çayönü PCA biplot (Fig. 8B) reflects a similar spread of measurements and a similar relationship between the variables (compare Fig. 8A,B). The archaeological charred *Triticum* measurements reflect the full spectrum of diploid and tetraploid wild and domesticated phenotypes, including wild-type diploid (*T. boeoticum*/*T. urartu*) and tetraploid wheat (e.g., *T. turgidum* spp. *dicoccoides*) alongside domesticated-type einkorn and emmer wheat (*T. monococcum*, *T. turgidum* ssp. *dicoccum*). Almost all the Gusir group specimens fall into the mid left-hand section of the plot, which indicates their status as wild-type einkorn with some likely representing 2-grained einkorn. A few specimens are located closer to the centre of the plot and may represent emmer grain. This is supported by the morphological observations of charred grain and chaff remains (Fig. 7C,E). The presence of tetraploid wheat at Gusir Höyük is also supported by the occurrence of tetraploid-type terminal spikelets (Fig. 7D). The Çayönü Phase Ia group contains a greater diversity of wild phenotypes, including both longer and shorter grains, most of which show a similar spread to the range of variability observed in the wild-type diploid modern *Triticum* accessions (Fig. 8A,B). Both Çayönü Phase Ia and Ib groups reflect greater variability in their measurements compared to the Gusir group. Some Phase Ib grains also show considerable overlap in their measurements with the PN Phase II group, which (as expected) overlaps with the range of domesticated-type einkorn and emmer grain (lower right-hand quadrant). This suggests that at least some of the Çayönü Phase Ib charred *Triticum* grains can be attributed to domesticated-type einkorn and emmer wheat.

## Discussion

The Gusir Höyük archaeobotanical assemblage provides the first conclusive evidence for the intensive use of crop progenitor taxa in the Anatolian Tigris basin during the early PPN. The results of our analysis indicate that plant exploitation focused on a narrow range of legume crop relatives (*Lens*, *Vicia/Lathyrus*, *Vicia ervilia*) alongside nuts (*Prunus* subg. *Amygdalus*, *Pistacia*) throughout the timespan represented by the sampled deposits (~ 11,400–10,300 cal BP) corresponding to the PPNA and EPPNB horizons. A set of closely controlled contexts dated to the EPPNB (~ 10,500–10,300 cal BP) contained the same range of legumes alongside a distinctive assemblage of cereal crop progenitors (1 and 2-grained einkorn and emmer grain and chaff, including a single einkorn grain AMS-dated to ~ 10,500 cal BP).

The available evidence suggests that during the PPNA legumes were most probably harvested from the wild. There is very little evidence for the exploitation (much less the cultivation) of riparian habitats, despite their predicted availability in the site environs considering also its location near the intersection of permanent watercourses. The absence of large-seeded grasses from the PPNA samples (and from the EPPNB samples of other large-seeded grasses associated with dryland habitats such as *Hordeum*) is notable, as is the absence from all sampled deposits of other legume crop relatives such as *Pisum* and *Cicer* spp. It is unlikely that this pattern could have been forced by preservation, taphonomic and/or sampling biases. It appears more probable that plant exploitation at Gusir Höyük was selective (rather than opportunistic) reflecting cultural preferences associated with plant use (e.g., site-specific harvesting routines, cooking practices and/or culinary choices) that were not overtly constrained by resource availability in the local environment. This is corroborated by the zooarchaeological results, illustrating a clear preference for species associated with dry (upland) habitats (partridges and caprines). Though few in numbers, the presence of water-bound taxa (beaver, crane, white-tailed eagle, fish, freshwater mussels) undisputedly proves that wetland habitats existed in the site environs. Future analyses will elucidate the degree to which faunal exploitation was subject to strictly seasonal resource scheduling or other cultural and/or environmental factors.

The picture of a narrow plant resource spectrum at Gusir Höyük, targeting a limited set of crop progenitors alongside nuts, is in stark contrast to the evidence available from other PPNA sites in the Tigris basin (Fig. 1). Archaeobotanical finds from Hallan Çemi and Demirköy indicate a diverse plant resource base including Brassicaceae, Chenopodiaceae and wetland plants (Cyperaceae, Polygonaceae) found alongside small-seeded grasses and Vicieae. Large-seeded legumes, barley and nuts are also present although they make up for a low proportion of the charred plant assemblage^2,34^. The use of a diverse plant spectrum is also attested at Körtik Tepe, where the available evidence points to a greater reliance on small-seeded grasses and legumes during its earliest phases (dated to the Younger Dryas) shifting to a broad range of other wild taxa, notably Cruciferae and Chenopodiaceae, at the start of the Holocene^3^. Cereal and legume crop progenitors contributed very little to sample composition, although this pattern may be somewhat exacerbated by context-related variation that remains unaccounted for in the published reports^35^. Further downstream, in the Jezireh and the Tigris basin of northwestern Iraq, the archaeobotanical assemblages of Qermez Dere and M’lefaat are dominated by a diverse spectrum of large-seeded legumes (Vicieae, *Lathyrus/Vicia*, *Lens*) and small-seeded grasses and legumes alongside Asteraceae and other wild taxa. Some large-seeded grasses including barley, einkorn and goatgrass are present in very low frequencies^2,34^. The only PPNA site potentially approximating the narrow spectrum of plant resources exploited at Gusir is Hasankeyf Höyük. Initial archaeobotanical results reported in summary form have hinted at the predominant presence of *Prunus* subg. *Amygdalus* and *Pistacia* nuts alongside *Celtis* fruits and a limited set of legume crop relatives (*Lens*, *Vicia ervilia*)^4,36^. However, unlike Gusir, the faunal spectrum exploited at Hasankeyf Höyük seems to have been as diverse as at other PPNA sites in the region^37^.

Overall, the regional archaeobiological record suggests that the PPNA communities of the Tigris basin pursued highly idiosyncratic plant and animal exploitation practices targeting plant “staples” that diverged markedly even between sites located in close proximity to each other. With regard to cereals, the Lake Van pollen data clearly show that Poaceae (including Cerealia) were present in southeast Anatolia at the start of the Holocene at levels equal to those observed at ~ 8000 cal BP, by which time farming in the region was well-established^12^. In the case of legumes, which are better represented in the sampled PPNA sites, no single site contains the full range of the taxa available in the regional vegetation. It is therefore highly unlikely that differences in local vegetation ecologies, or even the seasonal instability and periodic collapse of grassland habitats caused by a higher incidence of wildfires at the start of the Holocene^11^, could account for all or most of the variation observed in the archaeobotanical record. Instead, it is more plausible that this diversity was linked to distinctive local identities manifested in site-specific plant resource use and/or culinary choices. A comparable pattern of divergent resource choices characterizing aceramic Neolithic communities located in the same geographical area and exploiting similar ecotones has been previously detected in south-central Anatolia during the 12th–11th millennia cal BP^38^.

The plant resource spectrum reflected in the Gusir Höyük PPNA and EPPNB archaeobotanical assemblages (with its distinctive emphasis on crop progenitor taxa and nuts) is strongly reminiscent of the assemblages reported from Çayönü to the NW of the upper Tigris basin, and Göbeklitepe and Nevali Çori in the Euphrates basin^5–9^. What all these sites have in common is evidence for architectural complexity and symbolic/ritual elaboration. Göbeklitepe, Çayönü and Gusir are also characterized by long occupation sequences straddling the PPNA-EPPNB timespan^18,19,31,39^ . The composition of the Gusir Höyük EPPNB botanical assemblage points to the exploitation of a phenotypically wild crop progenitor mixture (including 1- and 2-grain einkorn and emmer wheat, *Lens*, *Vicia ervilia* and *Vicia*/*Lathyrus*) alongside the first appearance in the record of small-seeded legumes (*Medicago*, *Astragalus*, *Trigonella* and Fabaceae indet.) indicating increasing disturbance and/or possible herbivore grazing of the local vegetation during this period. The PCA of the morphometric characteristics of the Gusir *Triticum* spp. charred grains also points to the existence of a local wild-type wheat population, which was distinct from those represented in the Çayönü Phase Ia and Ib groups. Overall, considering also the PCA morphometric indicators for the presence of domesticated-type wheat grain in the Çayönü Phase Ib group, the combined radiometric, botanical and morphometric data from Gusir Höyük and Çayönü provide tangible indications for the existence in the upper Tigris basin of a regionally distinctive trajectory from the management of cereal crop progenitors in the early-mid 11^th^ millennium cal BP through to the first appearance of domesticated-type wheat sometime after ~ 10,300 cal BP.

To date, archaeobotanical evidence of a similar trajectory at contemporaneous EPPNB sites in the Anatolian Euphrates basin is equivocal. Nevali Çori (dated to ~ 10,500 cal BP) contained a diverse crop progenitor assemblage including 3 cereals (barley, einkorn and emmer wheat) alongside *Lens*, *Pisum*, *Vicia ervilia*, *V. faba*, *Cicer* and *Lathyrus*^9^. Hulled wheat remains were dominated by small-sized wild diploid and tetraploid *Triticum* spp. grains. Pasternak who studied this assemblage did not record systematically the wheat remains and did not measure individual grains, due to the low number of complete items found there^9^. His published grain drawings indicate the presence of small-sized wild-type einkorn, alongside a minority of domesticated-type einkorn and emmer wheat, in a pattern very similar to that detected by our PCA of the Çayönü Ib group. A later re-examination of 7,958 spikelet forks (from a total of 26,002 originally reported by Pasternak) indicated that most displayed signs of damage prior to charring, likely due to the dehusking of grain by pounding. Out of a total of 355 undamaged spikelet forks 243 were identified as dehiscent (wild-type), 39 as indehiscent (domesticated-type) and 73 as ‘possibly’ domesticated-type^27^. The abundance of chaff and the diversity of the crop progenitor spectrum suggest that cereals were almost certainly cultivated at Nevali Çori^9^. There are also at least some indications for the early onset of the domestication syndrome in hulled wheats (as suggested by the apparent co-occurrence of at least 2 different einkorn grain size-classes), despite the taphonomic bias (pounding damage) preventing the reliable detection of wild-/domesticated type chaff. The evidence from Göbeklitepe is even more ambiguous, due to the very limited recovery of charred plant macro-remains. Small-scale sampling (manual bucket flotation) of mixed material from the fill of the monumental buildings has revealed the presence of wild-type barley, rye/einkorn and einkorn grain and chaff^5^. However, more recently published microbotanical (phytolith) analyses have also indicated that domesticated-type barley and einkorn may be present^6^. If upheld by large-scale machine-assisted flotation sampling of closely controlled radiometrically-dated deposits (including the direct AMS dating of individual charred grain finds) and the analysis of a representative macrobotanical assemblage, these results alongside the Nevali Çori data may point to a slightly earlier start of cereal cultivation compared to Gusir, followed by the onset of phenotypic domestication in the Anatolian Euphrates basin during the EPPNB. Such a development could have been instigated by the close proximity of these sites to the communities further downstream in the Syrian Euphrates, where cereal crop progenitors were exploited during the PPNA^40^. Recent genomic research on the metabolite content of tetraploid wheats has revealed important differences in culinary and nutritional-related traits between wild and domesticated species, with the former being characterized by higher micronutrient content^41^. In turn, an important role of culinary traits in the intensification of cereal crop progenitor use during the EPPNB is compatible with the results of the present study as well as previous contextual analyses indicating the preferential use of cereals in communal food consumption and feasting at architecturally complex sites characterized by distinctive symbolic and ritual behaviors^42,43^.

The Gusir Höyük archaeobotanical assemblage, set in its regional context, calls into question current paradigms concerning the macro-evolutionary process of early cultivation and domestication in Southwest Asia. The available evidence of PPNA plant exploitation in southeast Anatolia does not support a protracted “pre-domestication cultivation” stage^11,42^ characterized by the intensive use and management of cereal crop progenitor species by sedentary communities. Despite the wide availability of cereals in the regional vegetation, at most sites they were neither “staples” nor is it possible to detect indicators of increasing human impacts on the landscape (e.g., a greater presence of ruderal taxa at PPNA Gusir Höyük). During the EPPNB, a diverse suite of cereal and legume crop progenitors appeared across several sites. Some (Nevali Çori, Çayönü) contain evidence of early domestication traits indicating selection pressures or bottlenecks impacting local populations of Triticeae (e.g., einkorn). The Gusir Höyük evidence shows that further east along the Tigris, locally available wild cereal populations were exploited after cereal cultivation was established along the Euphrates. The available evidence also suggests that initial cereal domestication in southeast Anatolia was probably more rapid than envisioned by the protracted transition hypothesis, being closely correlated in time and space with the intensification of animal management^17^. Further insights into the chronology and diversity of early crop progenitor use and the evolution of the domestication syndrome in southeast Anatolia are entirely dependent on the systematic retrieval and analysis of representative archaeobotanical assemblages from sites preserving long sequences spanning the PPNA-EPPNB horizon (e.g., Çayönü, Göbeklitepe) including the multivariate evaluation of grain morphometrics and the direct AMS dating of charred grain finds.

As exemplified by the Nevali Çori and Çayönü case studies, morphological observations of shattering habit (dehiscent/indehiscent chaff) and even less so linear increases in average seed size, do not constitute secure indicators of early cereal domestication or cultivation. This is because their manifestations in the archaeobotanical record depend on a multitude of taphonomic parameters and environmental factors other than human selection and agronomic conditions^10,11,26,27^. The results of our multivariate evaluation of archaeobotanical and modern *Triticum* metrics demonstrate that there is ample scope for future work in this field expanding to the morphometric identification of wheat remains to species/subspecies level, provided that measurements of individual grains (both complete and appropriately selected fragmentary specimens) are routinely collected and published. Our multivariate morphometric approach has provided detailed insights of diagnostic value into inter- and intra-assemblage grain size variation, which permit detecting wild-type and domesticated crop morphotypes in the Gusir Höyük, Çayönü and modern *Triticum* accessions. The low levels of grain size variation observed in the Gusir group, compared to the greater variability observed in the Çayönü Ia and Ib groups, likely reflect the existence at Gusir Höyük of a distinct, phenotypically wild, insular wheat population. Similarly detailed morphometric studies applied to other PPN charred grain assemblages hold great potential for the systematic examination of *Triticum* phenotypic variability before, during and after the crucial periods associated with the transition from foraging to farming in southeast Anatolia and across Southwest Asia. Further morphometric work including statistical shape analysis (geometric morphometrics) could also overcome the limitations imposed by high phenotypic plasticity in grain size and provide insights into possible inter- and intra-regional seed exchange routes and networks. Similar analyses may permit the archaeobotanical testing of genomic hypotheses about the importance of seed corn translocation and/or the exchange of mutant populations for the establishment and spread of domesticated einkorn cultivation in southeast Anatolia^44^. Finally, such analyses should be undertaken alongside the routine AMS dating of charred grain finds retrieved from closely controlled archaeological contexts. Together, they can revise and enhance in significant ways the timeseries (based on the per site/phase summed radiocarbon probability distributions) used by archaeobotanists for modelling the evolution of the classic traits (average seed size, shattering habit) associated with the domestication syndrome^45,46^.

## Methods

### Sampling and botanical identification

All flotation samples were collected and processed in the field by the excavation team during 2010–2014. Archaeobotanical flotation samples were subsequently passed through a stack of geological test sieves (meshes 2, 1, 0.5 and 0.25 mm) and sorted under a Leica S8APO stereozoom microscope (magnifications × 7- × 80) fitted with a GXCAM HICHROME-LITE HDMI 5MP camera connected to a laptop computer for the identification and recording of charred seed, chaff, fruit and nutshell remains. Wood charcoals > 2 mm were identified using a Brunel SP-110 M reflected-light (BF/DF) modular metallurgical microscope (magnifications × 50- × 500) along the 3 anatomical planes (TS = Transverse Section, RLS = Radial Longitudinal Section and TLS = Tangential Longitudinal Section). The wood anatomical distinction drawn between *Prunus* subgenus *Amygdalus* (ring-porous; ray width 3–5 or 6–8[10]seriate) and *Prunus* spp. including plums, cherries, etc. (diffuse to semi-ring porous; pores arranged mostly in radial files; rays predominantly narrow 1–3 or 3-5seriate) has its basis on modern wood anatomical studies^47^ and has been extensively verified by anthracological studies across Southwest Asia and the Eastern Mediterranean^48^. It also agrees with previous and recent botanical and genetic work in the region that separates *Amygdalus* as a distinct subgenus within the newly classified *Prunus*^49^. Both wood and non-wood charred macrofossils were identified by comparison to published works^33,47^ and materials available at the Department of Archaeology, Classics and Egyptology (University of Liverpool) and the Institute of Archaeology (UCL). These were subsequently verified through the multivariate (PCA) analysis of grain morphometric data (PCA results presented in the paper; see also Fig. 8, Supplementary Data Files S3-S7). Chaff was identified, where possible, to species-level by comparison to *Triticum* accessions made available by the John Innes Centre GRU (Norwich, UK). The archaeofaunal remains were taxonomically identified applying standard zooarchaeological methods. Bird and fish bones were identified with the aid of the reference collection of the Staatssammlung für Anthropologie und Paläoanatomie München.

### Quantification of non-wood charred plant macrofossils

Sorting of non-wood charred plant remains was carried out in full for all > 1 mm fractions, including the identification and quantification of all complete and fragmentary remains. In the < 1 mm fractions only items preserving a sufficient number of diagnostic features were sorted, in order to obtain secure botanical identifications. For charred nutshell, both fragment counts and weights are reported (calculating whole equivalents was not possible for the Gusir Höyük charred nutshell taxa). Wild-type Poaceae seed counts represent whole grain equivalents, which were calculated by combining the counts of whole grains and either the apical end or the embryo counts of fragmentary remains (whichever was greater) following commonly applied methodologies in Southwest Asian archaeobotanical research^50^. Fragmentary Poaceae seeds lacking apical or embryo ends (i.e., body fragments or laterally split seeds) are reported separately as ‘fragment counts’. All other non-wood charred plant counts (e.g., wild seeds, legumes) represent complete seed counts, with fragmentary remains reported separately (Supplementary Table S1, Supplementary Data File S2).

### *Triticum* grain morphometrics

Grain morphology (hence grain size measurements) can be greatly affected by charring conditions^51–53^. Charring under high-temperature (> 250 °C) conditions can result in severe distortion of grain shape and the appearance of protrusions due to the starchy endosperm ‘spilling out’ of the testa and pericarp. Some authors also report distortion of grain width^52^. No such distortions were observed in the great majority of the Gusir Höyük *Triticum* grain specimens while several specimens also contained better-preserved testa (Fig. 7F,G), all of which suggest that the Gusir Höyük grains were charred under relatively low temperatures. A minority of specimens displayed evidence of pericarp and testa lifting/loss (Fig. 7H). This likely represents damage to the outer layers of the grain caused by crop processing (e.g., dehusking by pounding) particularly since it is not accompanied by burning-related distortions such as bulbous protrusions of starchy endosperm and grain puffing. All measurements were carried out on whole and fragmentary grains displaying features of low-temperature charring (including well-preserved epidermal layers) in order to ensure that the measured items were not affected by burning-related distortions of their dimensions.

3 different measurements were carried out on all measured specimens (including modern *Triticum* accessions and the Gusir Höyük charred grains) following established protocols^33,54^. Length was measured from the apical end to the embryo end of the grain in the ventral view, excluding the protruding embryo (when present). Breadth was measured on the widest part of the ventral view from one lateral side of the grain to the other. Height was measured at the widest point of the lateral view from the ventral to the dorsal face of the grain. The Çayönü *Triticum* grain measurements were sourced from the published archaeobotanical report^7^. The full measurements datasets (from Gusir Höyük, Çayönü and the modern *Triticum* accessions) are included in Supplementary Data Files S3-S5.

### Multivariate analysis

Principal Component Analysis (PCA) on the measurements of modern *Triticum* accessions and the archaeological specimens was carried out using R version 4.0.2, package FactoMineR^55,56^. The resulting PCAs were plotted using the factoextra package, including drawing of point concentration ellipses at 0.9 for pre-defined groups (Fig. 8). The full PCA results including eigenvalues, variable contributions to dimensions and cos2 are presented in Supplementary Data Files S6-S7. The missing values in the Gusir Höyük grain measurement dataset (incomplete grains) were imputed using the missMDA package^57,58^. The imputed values to replace the missing observations (i.e., missing length measurements on incomplete specimens) were drawn from a gaussian distribution with mean and standard deviation calculated from the observed values (i.e., the entire range of length measurements from Gusir Höyük and Çayönü). In order to maintain the integrity of the dataset, imputation of the missing values was carried out only on charred grains from Gusir with incomplete length measurements (e.g., those having complete breadth and height measurements). The mean, minimum and maximum values and the standard deviations of the length, breadth and height measurements for each modern accession and the archaeological specimen groups are listed in Supplementary Data File S8. The ranges of values obtained for the Gusir specimens demonstrate that the imputed length measurements are within an acceptable standard deviation of the raw dataset of 7 measurements on complete grains (standard deviation of 0.35 mm, range 4.2–5.24 mm) and the imputed dataset of 20 grains (standard deviation of 0.31 mm, range 4.2–5.37 mm). The finalized imputed dataset (Supplementary Data File S5) is one which gives the smallest mean square error between the fitted and observed (original) matrices, also taking into account the relationships in the archaeological dataset between length, height and breadth.

## Supplementary Information

Supplementary Information 1.

Supplementary Information 2.

Supplementary Information 3.

Supplementary Information 4.

Supplementary Information 5.

Supplementary Information 6.

Supplementary Information 7.

Supplementary Information 8.

Supplementary Information 9.

## Data Availability

All data generated and analysed during this study are included in this published article (and its Supplementary Information files).
