# Supplementary material for: Pathways to plant domestication in Southeast Anatolia based on new data from aceramic Neolithic Gusir Höyük
Source: Sci Rep. 2021 Jan 22;11:2112. doi: 10.1038/s41598-021-81757-9 (PMC7822836; doi:10.1038/s41598-021-81757-9)
Supplement: Supplementary file 1 — Supplementary Information 1. [file 41598_2021_81757_MOESM1_ESM.pdf]

Supplementary Information

Pathways to plant domestication in Southeast Anatolia based on new data from aceramic Neolithic Gusir Höyük

Ceren Kabukcu[1,*], Eleni Asouti[1], Nadja Pöllath[2], Joris Peters[2,3] & Necmi Karul[4]

[1] University of Liverpool, Department of Archaeology, Classics and Egyptology, Liverpool, L69 7WZ, UK.
[2] Staatliche Naturwissenschaftliche Sammlungen Bayerns, Staatssammlung für Anthropologie und Paläoanatomie München, Karolinenplatz 2a, Munich, 80333, Germany.
[3] Ludwig-Maximilians-Universität München, ArchaeoBioCenter and Institute of Palaeoanatomy, Domestication Research and the History of Veterinary Medicine, Munich, 80539, Germany.
[4] Istanbul University, Department of Prehistory, Istanbul, 34134, Turkey.

[*]C.Kabukcu@liverpool.ac.uk

This PDF file includes:

Supplementary Figure S1
Supplementary Tables S1-S2

Additional supplementary materials for this manuscript include the following:

Supplementary Data Files S1 to S8

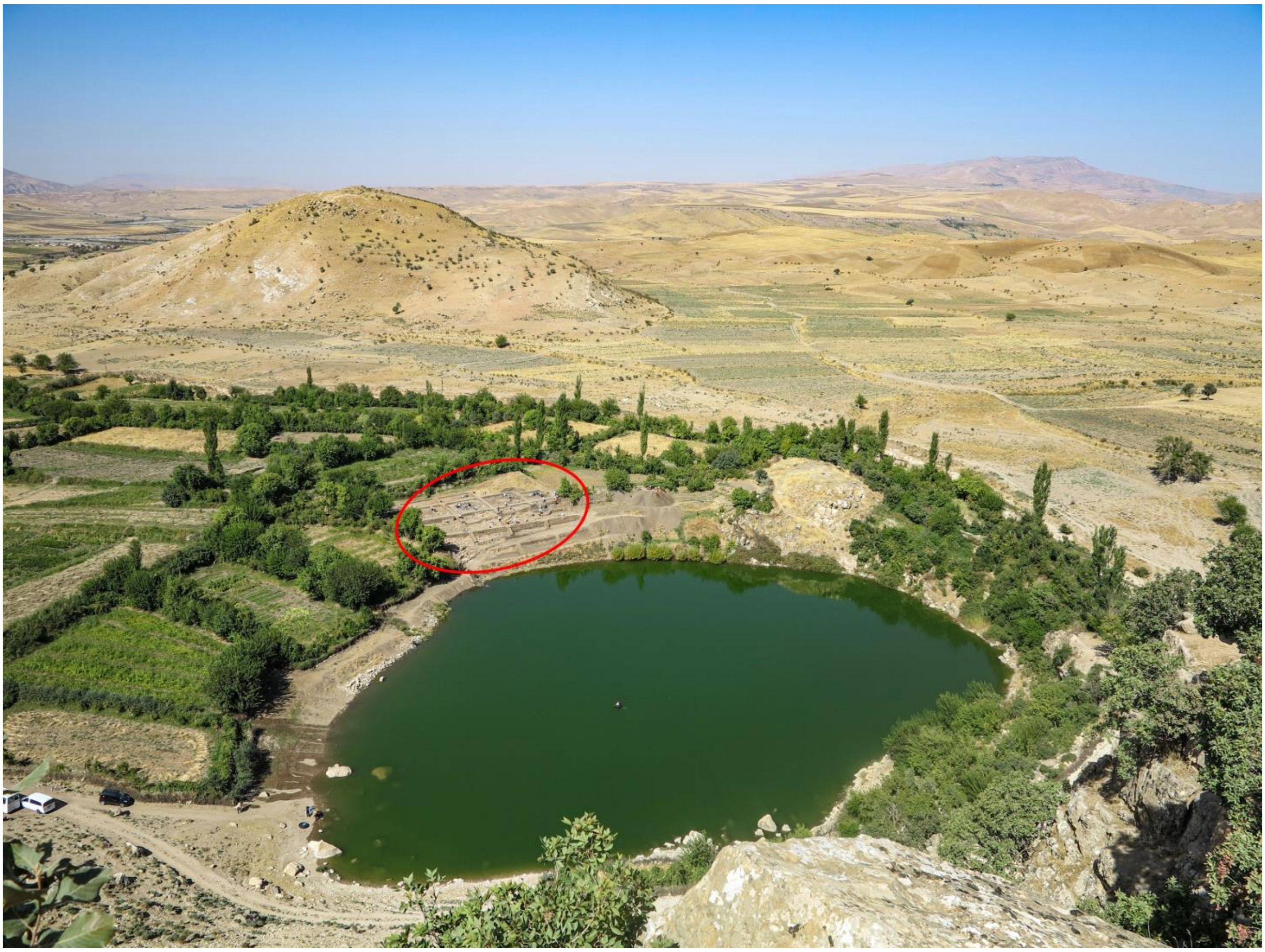

Supplementary Fig. S1. Photo showing the location of the excavated area (red circle) at Gusir Höyük next to the Gusir sinkhole lake (photo from the Gusir Höyük project archive).

Supplementary Table S1. Per phase counts of the charred plant macro-remains studied from Gusir Höyük: ~11.4-10.9 kyr cal BP group corresponds to the PPNA; ~10.5-10.3 kyr cal BP group corresponds to the EPPNB.

	**~11.4-10.9 kyr cal BP**	**~10.5-10.3 kyr cal BP**		**Total**
**Excavation grid square**	**20-M, N; 21-G, L, N; 22-J; 23-K, L**	**23-M**	**20-I**	
**Context type**	**Building fills**	**Ashy fills**	**Fire pits**	
**No of sampled contexts (= no of samples)**	**27**	**4**	**5**	**36**
**Nutshell & *Celtis***				
Prunus subg. *Amygdalus* nutshell (fragment count)	875	8	258	**1141**
Prunus subg. *Amygdalus* nutshell weight (g)	2.512	0.017	0.273	**2.802**
Pistacia nutshell (fragment count)	383	25	289	**697**
Pistacia nutshell weight (g)	0.56	0.041	0.154	**0.755**
Nutshell indet. (fragment count)	386		117	**503**
Celtis	2	3	4	**9**
**Fabaceae**				
Lens spp. (whole, cotyledon count)	113	8	20	**141**
Vicia cf. *ervilia* (whole, cotyledon count)	51	13	8	**72**
Vicieae indet. (whole, cotyledon count)	31	2	2	**35**
Vicieae indet. (fragment count)	18		8	**26**
Onobrychis cf. *viciifolia* (whole, cotyledon count)	4			**4**
Vicia/Lathyrus (whole, cotyledon count)	184	24	20	**228**
Vicia/Lathyrus (fragment count)	45		7	**52**
Medicago radiata		14	1	**15**
Astragalus/Trigonella		10	1	**11**
Fabaceae (small)		3	4	**7**
Fabaceae indet. (fragment count)	262	31	91	**384**
**Poaceae**				
Triticum boeoticum (wide) (whole + apical/embryo)		34		**34**
T. boeoticum (wide) (fragment count)		2		**2**
T. boeoticum (narrow) (whole + apical/embryo)		13		**13**
T. turgidum spp. *dicoccoides* (whole + apical/embryo)		14		**14**
T. turgidum spp. *dicoccoides* (fragment count)		3		**3**
Triticum spp. (fragment count)		207		**207**
T. boeoticum chaff (spikelet fork count)		3		**3**
T. boeoticum chaff (glume base count)		9		**9**
T. turgidum spp. *dicoccoides* chaff (glume base count)		1		**1**
Triticum spp. chaff (spikelet fork count)		21		**21**
Triticum spp. chaff (glume base count)		24		**24**
Aegilops spp. grain (whole + apical/embryo)		2		**2**
Aegilops spp. chaff		3		**3**
Poaceae wild (small) grain (whole + apical/embryo)	16		6	**22**
Poaceae wild (small) (fragment count)	75	16	11	**102**
Poaceae wild (medium) grain (whole + apical/embryo)	1		5	**6**
Poaceae indet. (fragment count)	12	4	28	**44**
Poaceae culm	2	12	3	**17**

**Wild/weedy other**				
Gypsophila	12	1	2	**15**
Silene	1			**1**
Brassicaceae	14			**14**
Brassica/Sinapis	29			**29**
Neslia	1			**1**
Apiaceae (cf. *Lisea*)	1			**1**
Lamiaceae	2	4		**6**
Galium	1			**1**
Cyperaceae (*Bolboschoenus/Carex*)	7	3	4	**14**
Adonis		1		**1**
Boraginaceae			1	**1**
Vitis sylvestris			1	**1**
Tuber/rhizome indet.	5			**5**
**Non-wood NISP**	**2533**	**518**	**891**	**3942**
Cupule/Involucre fragment	4			**4**
Amorphous charred plant/aggregate	67		15	**82**
**Wood charcoal >2mm**				
Prunus subg. *Amygdalus*	378		15	**393**
Pistacia	96	14	190	**300**
Prunus	3			**3**
Quercus	4	12	10	**26**
Betula	10	84	3	**97**
Acer	10		7	**17**
Maloideae			2	**2**
Rhamnus	4			**4**
Ulmaceae/cf. *Celtis*	1		1	**2**
Fraxinus	2	160	1	**163**
Alnus			1	**1**
Chenopodiaceae	1			**1**
**Wood charcoal >2mm NISP**	**509**	**270**	**230**	**1009**

Supplementary Table S2. Preliminary zooarchaeological results from PPNA contexts at Gusir Höyük, showing taxa with clear habitat preferences (NISP=Number of Identified Specimens; Habitat codes: M=mountain; G=Grassland; W=woodland; Wet/Aq=wetland/aquatic).

**Habitat**	**Taxon**	**NISP**
M	Wild goat, *Capra aegagrus*	334
G	Wild sheep, *Ovis orientalis*	768
G	Brown hare, *Lepus europaeus*	3
G	Chukar & Grey partridge, *Alectoris chukar* & *Perdix perdix*	438
W	Aurochs, *Bos primigenius*	327
W	Red deer, *Cervus elaphus*	1392
W	Roe deer, *Capreolus capreolus*	19
W	Wild boar, *Sus scrofa*	501
W	Brown bear, *Ursus arctos*	5
Wet/Aq	Beaver, *Castor fiber*	3
Wet/Aq	Common crane, *Grus grus*	1
Wet/Aq	White-tailed eagle, *Haliaeetus albicilla*	1
Wet/Aq	Fish	126
Wet/Aq	*Unio* shell	7

Supplementary Data File S1 (.xlsx file). Radiocarbon dates from the Gusir Höyük excavation grid squares sampled for charred plant remains.

Supplementary Data File S2 (.xlsx file). Counts of charred plant remains retrieved from each sampled excavation grid square at Gusir Höyük, grouped by chronological phase and context type.

Supplementary Data File S3 (.xlsx file). Measurements of *Triticum* accessions - All specimens sourced from the John Innes Centre Germplasm Resource Unit (GRU) (Norwich, UK).

Supplementary Data File S4 (.xlsx file). Archaeological charred *Triticum* measurements from Gusir Höyük (GSR) and Çayönü (CAY).

Supplementary Data File S5 (.xlsx file). Archaeological charred *Triticum* grain measurements used in PCA, including missMDA imputed length measurements of incomplete Gusir Höyük grains.

Supplementary Data File S6 (.xlsx file). PCA results of the *Triticum* accessions measurements.

Supplementary Data File S7 (.xlsx file). PCA results of the archaeological charred *Triticum* measurements.

Supplementary Data File S8 (.xlsx file). Mean, minimum and maximum values and standard deviations of length, breadth and height measurements for each modern *Triticum* accession and the archaeological specimen groups.
